# Exploring Mixed Feeding Practices Among Indian Mothers: Knowledge, Attitudes, and Reported Barriers to Exclusive Breastfeeding

**DOI:** 10.7759/cureus.109092

**Published:** 2026-05-18

**Authors:** Umamah Hassan, Sangita Yadav, Neha Bansal, Sana Khan, Rekha Harish

**Affiliations:** 1 Pediatrics, Hamdard Institute of Medical Sciences and Research, New Delhi, IND; 2 Pediatrics, School of Medical Sciences and Research, Greater Noida, IND; 3 Pediatrics and Child Health, Hamdard Institute of Medical Sciences and Research, New Delhi, IND

**Keywords:** antenatal care, bottle feeding, breastfeeding, lactation counselling, mixed feeding

## Abstract

Background

Mixed feeding, the practice of combining breastfeeding along with formula or other foods/liquids before six months of age, is a common concern in India. This study aims to explore the factors influencing mixed feeding among Indian mothers, focusing on their knowledge, attitudes, and practices. Understanding these factors can help in designing targeted interventions to promote exclusive breastfeeding (EBF) as per World Health Organization recommendations.

Methodology

We conducted a hospital-based observational study that included 129 mothers practicing mixed feeding. A questionnaire assessed their knowledge, attitudes, and determinants related to breastfeeding.

Results

While most mothers (95.3%) understood the importance of EBF for the first six months, challenges such as inadequate milk supply (76.7%), family influence, and workplace constraints led to mixed feeding. Formula and cow’s milk were the most used alternatives.

Conclusions

The study underscores the need for improved lactation counselling and support, especially during antenatal and postnatal care. Strengthening public health interventions and workplace policies can help promote EBF.

## Introduction

Infant and young child feeding (IYCF) is a critical determinant of child health and survival, particularly during the first two years of life. Breastfeeding, especially exclusive breastfeeding (EBF) during the first six months, provides optimal nutrition and is one of the most cost-effective interventions for reducing infant mortality and morbidity worldwide. Breast milk contains essential nutrients, immunological components, and growth factors that protect against common childhood infections such as pneumonia, meningitis, diarrhea, and neonatal sepsis, which are major contributors to infant mortality in developing countries [1]. Evidence suggests that EBF significantly reduces the risk of these infections and improves overall infant health outcomes, with the potential to prevent a substantial proportion of infant deaths [2]. In addition to its nutritional and immunological benefits, breastfeeding promotes maternal-infant bonding and contributes to maternal well-being, further underscoring its importance [3].

Despite these well-established benefits, EBF rates remain suboptimal in many developing countries, including India. Mixed feeding, wherein infants receive both breast milk and supplementary feeds such as formula, remains a common practice despite global recommendations advocating EBF for the first six months. This practice reduces the protective benefits of breastfeeding and increases the risk of infections [4]. Although institutional delivery rates in India have improved significantly, reaching over 88% as reported in the fifth National Family Health Survey (NFHS-5) (2019-2021), only 64% of infants are exclusively breastfed for the first six months, highlighting a persistent gap between healthcare utilization and optimal infant feeding practices [5]. This gap represents a significant public health concern and underscores the need for targeted interventions.

To address this issue, several government initiatives, including the Baby-Friendly Hospital Initiative, Janani Shishu Suraksha Yojana, and the Mother’s Absolute Affection program, have been implemented to promote breastfeeding and improve IYCF practices. These programs aim to educate mothers and healthcare providers about optimal feeding practices, particularly EBF. However, barriers such as limited maternal education, cultural beliefs, and lack of awareness continue to hinder the effectiveness of these initiatives [6]. Understanding mothers’ knowledge, attitudes, and practices regarding breastfeeding is essential for designing effective interventions to improve breastfeeding rates and child health outcomes in India [7].

The present study was undertaken to explore the factors influencing mixed feeding practices and to identify barriers to EBF during the first six months of life. It also aimed to assess the knowledge and attitudes of mothers regarding EBF. The primary objective was to assess the knowledge and attitudes of mothers regarding EBF. The secondary objective was to identify determinants of mixed feeding and barriers to EBF during the first six months of life.

## Materials and methods

The study was conducted after obtaining ethical approval from the Institutional Ethical Review Committee (approval number: HIMSR/IEC/0074/2022). A total of 129 women attending the neonatal clinic or admitted to the pediatric ward of a tertiary care center and on mixed feeding participated after obtaining the informed consent. The study was conducted to assess the determinants of non-EBF and evaluate maternal knowledge, attitudes, and practices related to EBF for infants aged six weeks to six months.

Mothers of term, appropriate for gestational age infants attending the immunization clinic, following up in the neonatal clinic, or admitted to the pediatric ward of a tertiary care medical institute were approached for participation. Written informed consent was obtained from those who agreed to participate. Only mothers of infants on non-EBF (i.e., mixed feeding) were included in the study. Infants who were preterm, small for gestational age, had major congenital anomalies, or whose mothers were on contraindicated medications (such as antipsychotics or chemotherapy) were excluded from the study to ensure that the sample represented healthy, term infants. Mixed feeding was defined as the practice of providing breast milk along with formula milk, animal milk, or other supplementary feeds before six months of age.

The study period was three years. Participants were recruited using convenience sampling. The questionnaire was administered in Hindi/local language whenever required. The sample size was calculated using the formula:

\begin{equation} n = \frac{Z^2 \times P \times (1 - P)}{e^2} \label{eq:placeholder_label} \end{equation}

where Z = 1.96 at a 95% confidence interval, P = 0.64 (prevalence of EBF as per NFHS-5), and e = 0.08 (absolute precision). The minimum required sample size was 129.

All mothers whose infants were found to be non-exclusively breastfed were provided with counselling on the importance of EBF, and efforts were made to encourage a return to exclusive breastfeeding practices. The confidentiality of the participants was maintained throughout the study.

Data collection was performed through a structured interview schedule, which was developed to assess various aspects of maternal knowledge and breastfeeding practices. Items for the knowledge and attitude of EBF scales of the questionnaire were adapted from the Food and Agriculture Organization of the United Nations (FAO) guidelines for assessing nutrition-related knowledge, attitudes, and practices manual [8]. The interviews were conducted in the local language to ensure that the participants fully understood the questions. The interview schedule consisted of maternal and infant profiles, knowledge and attitude assessment, and breastfeeding practices. Efforts were made to minimize recall and interviewer bias by using a structured, pre-tested questionnaire and conducting interviews in the local language.

The data were analysed using descriptive statistics to summarize the demographic and feeding characteristics. Statistical analysis was conducted using SPSS software version 25.0 (IBM Corp., Armonk, NY, USA). As the study was descriptive in nature, data analysis was limited to frequencies and percentages. No inferential statistical tests or hypothesis testing were performed.

## Results

The study assessed mothers’ knowledge and attitudes toward breastfeeding and the determinants of non-EBF during the first six months of life. A total of 129 mothers were enrolled. Data regarding knowledge and attitudes were collected using a preformed questionnaire.

Among the participants, the majority (61, 47.3%) were in the age group of 26-30 years, followed by 40 (31%) in the 20-25-year age group, while 27 (20.9%) were above 30 years of age. Regarding education, 41 (31.9%) mothers were graduates, and 36 (28.3%) had completed high school. Most mothers (113, 87.6%) were housewives, while 15 (11.6%) were employed. Approximately 103 (80%) families had a monthly income of more than INR 7,500.

With respect to obstetric characteristics, 73 (56.6%) mothers were primigravida and 56 (43.4%) were multigravida. Vaginal delivery was reported in 81 (62.8%) mothers, while 48 (37.2%) had undergone caesarean section.

Mothers’ knowledge regarding breastfeeding is presented in Table 1. A high proportion of mothers were aware that EBF is important (123, 95.3%) and that breastfeeding should be initiated within one hour of birth (119, 92.2%). Most mothers (114, 88.4%) correctly understood that no food or water should be given during the first six months, and 107 (82.9%) were aware that water should not be given before six months. Knowledge regarding colostrum was also high, with 128 (99.2%) recognizing its nutritional benefits. Additionally, 92 (71.3%) mothers were aware that prelacteal feeds should not be given, and 107 (82.9%) believed that galactagogues can improve milk production. Despite this high level of awareness, only 30 (23.3%) mothers practiced EBF for the first six months.

**Table 1:** Mothers’ knowledge regarding breastfeeding.

Knowledge	True, n (%)	False, n (%)
Is exclusive breastfeeding important	123 (95.3%)	6 (4.7%)
Initiation within 1 hour	119 (92.2%)	10 (7.8%)
Water before 6 months	22 (17.1%)	107 (82.9%)
Only breastmilk (no food/water)	114 (88.4%)	15 (11.6%)
Medicines allowed	125 (96.9%)	4 (3.1%)
Colostrum beneficial	128 (99.2%)	1 (0.8%)
Prelacteal feeds avoided	92 (71.3%)	37 (28.7%)
Galactagogues improve milk	107 (82.9%)	22 (17.1%)

The attitudes of mothers toward breastfeeding are shown in Table 2. A majority of mothers agreed that breastfeeding should be on demand (107, 82.9%) and continued up to two years of age (95, 73.6%). Most mothers (93, 72.1%) disagreed with stopping breastfeeding during episodes of diarrhea. While only 9 (7%) believed that formula feeding is better than breastfeeding, a considerable proportion (77, 59.7%) felt that formula feeding is a more practical option when the mother returns to work. Additionally, 34 (26.4%) mothers reported a belief in giving prelacteal feeds.

**Table 2:** Attitudes of mothers toward breastfeeding.

Attitude	Agree, n (%)	Disagree, n (%)	Unsure, n (%)
On-demand feeding	107 (82.9%)	8 (6.2%)	14 (10.9%)
Continue till 2 years	95 (73.6%)	4 (3.1%)	30 (23.3%)
Prelacteal feeds	34 (26.4%)	60 (46.5%)	35 (27.1%)
Stop breastfeeding during diarrhea	21 (16.3%)	93 (72.1%)	15 (11.6%)
Formula better	9 (7%)	98 (76%)	22 (17.1%)
Formula if working	77 (59.7%)	33 (25.6%)	19 (14.7%)
Missing motherhood joy	113 (87.6%)	7 (5.4%)	9 (7%)

The determinants of mixed feeding are presented in Table 3. The most common reason was perceived inadequate breast milk supply or the belief that the baby remained hungry (99, 76.7%). Other contributing factors included busy lifestyles (7, 5.4%), discomfort with feeding in public (7, 5.4%), breast problems (9, 7%), maternal surgery (2, 1.6%), night feeds (1, 0.8%), need for a break from breastfeeding (2, 1.6%), and difficulty in latching (2, 1.6%).

**Table 3:** Determinants of mixed feeding.

Cause	n (%)
Inadequate milk/hungry baby	99 (76.7%)
Busy lifestyle	7 (5.4%)
Uncomfortable in public	7 (5.4%)
Breast problems	9 (7%)
Maternal surgery	2 (1.6%)
Night feeds	1 (0.8%)
Need for a break	2 (1.6%)
Latching difficulty	2 (1.6%)

Family influence played a significant role in feeding decisions (Table 4). The most common sources of advice for initiating bottle feeding were mothers-in-law (58, 45%) and husbands (44, 34.1%), followed by doctors (12, 9.3%). A small proportion of mothers reported influence from media (4, 3.1%), sisters (4, 3.1%), or their own decision (7, 5.4%).

**Table 4:** Source of advice for bottle feeding.

Source	n (%)
Husband	44 (34.1%)
Mother-in-law	58 (45%)
Doctor	12 (9.3%)
Media	4 (3.1%)
Sister	4 (3.1%)
Own decision	7 (5.4%)

Healthcare support was variable among participants. Approximately 74 (57%) mothers reported receiving breastfeeding advice during antenatal visits, while only 39 (30%) received postnatal support.

## Discussion

Although India has witnessed a substantial rise in institutional deliveries in recent years, reaching over 88% as per NFHS-5 (2019-2021), the proportion of infants exclusively breastfed for the first six months remains comparatively low at 64%, highlighting a persistent gap between healthcare access and optimal infant feeding practices [5].

The present study also reflects this gap. Despite a high level of awareness regarding breastfeeding practices among mothers, the proportion practicing EBF for the recommended six months remained low at 30 (23.3%), highlighting a significant gap between knowledge and actual practice. Mixed feeding remains a common practice, with several contributing factors. In this study, the perception of inadequate breast milk was the most frequently reported reason for initiating mixed feeding, observed in 99 (76.7%) mothers. Other factors included breastfeeding difficulties such as pain or latching issues, breast problems (9, 7%), and lifestyle-related challenges such as busy schedules (7, 5.4%).

Mode of delivery has been shown to influence breastfeeding practices. Hulman et al. [9] reported that 67.6% of mothers who underwent cesarean section opted for bottle feeding. Similar findings have been observed in a meta-analysis by Gebenyahu et al. [10], where EBF rates were lower among cesarean-delivered infants, possibly due to factors such as postoperative pain, delayed initiation, and prolonged hospital stay.

The perception of insufficient breast milk, identified as the leading cause of mixed feeding in this study, is consistent with findings from other studies, such as Chinnasami et al. [11] and Sultania et al. [12]. This perception, however, is often not based on actual physiological insufficiency but rather on misinterpretation of infant cues. Studies have shown that appropriate lactation counselling and postnatal support can effectively address these concerns and reduce unnecessary supplementation, as highlighted by Makwana [13]. A systematic review by Montero et al. [14] also emphasized that maternal concerns regarding infant satiety and milk adequacy are major drivers of mixed feeding practices, particularly in the early months of life.

Family influence emerged as another important determinant of feeding practices in this study. Mothers-in-law (58, 45%) and husbands (44, 34.1%) were the most common sources of advice for initiating bottle feeding. Similar findings have been reported by Makwana [13], where 31.8% of mothers introduced bottle feeding based on family advice. These findings underscore the importance of involving family members in breastfeeding education and counselling to ensure consistent and supportive messaging.

Occupational factors also played a significant role. In the present study, 38 (29.4%) mothers reported returning to work as a reason for initiating mixed feeding. This is in line with findings by Kumar and Mundhra [15], who observed a shift toward bottle feeding among working mothers due to inadequate maternity leave and lack of workplace support. Similarly, Hazir et al. [16] reported that insufficient maternity leave and the absence of breastfeeding-friendly workplace environments negatively impact EBF rates. These findings highlight the need for supportive workplace policies, including extended maternity leave, flexible working conditions, and the provision of lactation facilities.

In terms of feeding practices, formula milk (60, 46.6%) and cow’s milk (58, 45%) were the most commonly used alternatives to breast milk, reflecting an increasing reliance on supplementary feeding options. Similar trends have been reported by Feleke et al. [17]. While these alternatives may offer convenience, they are associated with increased risks of infections and adverse health outcomes, particularly in resource-limited settings [11].

Despite high awareness regarding EBF, the actual practice remained low, suggesting that knowledge alone may not be sufficient to ensure adherence to recommended feeding practices. Sociocultural influences, perceived inadequate milk supply, family pressure, and occupational constraints may contribute to this knowledge-practice gap.

Study limitations

This study has certain limitations. Being a hospital-based study, the findings may not be generalizable to the wider community. The cross-sectional design limits the ability to establish causal relationships. Additionally, the data were self-reported and may be subject to recall bias and social desirability bias. Additionally, the assessment of insufficient milk supply was based on maternal perception and was not objectively verified clinically. The relatively small sample size may limit the generalizability of the findings.

However, despite these limitations, the study provides valuable insights into breastfeeding practices and determinants of mixed feeding in an urban tertiary care setting, which may help inform targeted interventions. As this was a cross-sectional observational study without a control group, causal relationships between individual determinants and feeding practices could not be established.

## Conclusions

While mothers in the mixed feeding group were knowledgeable about breastfeeding, misconceptions about milk supply, familial pressures, and work-related barriers contributed significantly to the adoption of mixed feeding. Addressing these issues through improved lactation support and targeted education campaigns could reduce the prevalence of mixed feeding and promote EBF, particularly in the critical first six months of life.

## Appendices

Study questionnaire

**Table 5:** Questionnaire.

Section	Serial number	Variable/Question	Response options
Sociodemographic characteristics	1	Age (years)	<19/20–25/26–30/>30
	2	Income (INR/month)	<2,500/2,501–5,000/5,001–10,000
	3	Marital status	Married/Unmarried
	4	Religion	Hindu/Muslim/Christian/Others
	5	Education	Illiterate/Primary/Secondary/Undergraduate/Postgraduate
	6	Type of delivery	Normal/Caesarean
	7	Gravida	Primigravida/Multigravida
	8	Current breastfeeding	Yes/No
	9	Exclusive breastfeeding	Yes/No
	10	Initiation within 1 hour	Yes/No
Knowledge of mothers	11	Colostrum is the first breast milk	True/False
	12	Colostrum is important for immunity	True/False
	13	Burping should be done after each feed	True/False
	14	Breastfeeding should continue up to 2 years	True/False
	15	Exclusive breastfeeding should be given for 6 months	True/False
	16	A healthy diet improves milk production	True/False
	17	The mother should sit comfortably while feeding	True/False
	18	Eye contact and interaction during feeding are important	True/False
	19	Breasts should be washed before each feed	True/False
	20	The baby should be awakened for feeding if required	True/False
	21	Breastfeeding promotes bonding	True/False
	22	Breastfeeding prevents breast diseases	True/False
	23	Breastfeeding should be stopped during diarrhoea	True/False
	24	Breastfeeding should stop when weaning starts	True/False
Attitudes of mothers	25	Benefits last only while breastfeeding continues	Agree/Disagree/Unsure
	26	Formula feeding is more convenient	Agree/Disagree/Unsure
	27	Breastfeeding improves bonding	Agree/Disagree/Unsure
	28	Breast milk lacks iron	Agree/Disagree/Unsure
	29	Colostrum should be discarded	Agree/Disagree/Unsure
	30	Prelacteal feeding should be given	Agree/Disagree/Unsure
	31	Formula-fed babies are more likely to be overfed	Agree/Disagree/Unsure
	32	Formula feeding is better if the mother works	Agree/Disagree/Unsure
	33	Formula-feeding mothers miss an important experience	Agree/Disagree/Unsure
	34	Women should not breastfeed in public	Agree/Disagree/Unsure
	35	Breastfed babies are healthier	Agree/Disagree/Unsure
	36	Breast milk is an ideal food	Agree/Disagree/Unsure
	37	Breast milk is easier to digest	Agree/Disagree/Unsure
	38	Breastfeeding is more convenient	Agree/Disagree/Unsure
	39	Breastfeeding is cheaper than formula feeding	Agree/Disagree/Unsure
Determinants of bottle/mixed feeding	40	Time of initiation of bottle feeding	Soon after birth/Initially BF then switched (specify duration)
	41	Type of milk used	Expressed breast milk/Cow milk/Buffalo milk/Formula milk
	42	Dilution practice	No dilution/1:1/2:1
	43	Reason for stopping breastfeeding	Weaning/Inadequate milk/Public discomfort/Return to work/Lack of support
	44	Reason for mixed feeding	Weaning/Break/Inadequate milk/Busy lifestyle/Night feeds
	45	Reason for exclusive bottle feeding	Previous experience/Public discomfort/Husband support/Other children/Low milk/Failed BF/Work constraints/Measure intake
	46	Feeding method in the previous child	Breastfeeding/Bottle feeding/Mixed
	47	Guidance for formula preparation	Yes (specify source)/No
	48	Preparation method	Fresh each time/Prepared and stored
	49	Frequency of formula feeding	Almost all feeds/Half feeds/1–2/day/Few/week
